# Strategies used by the Canadian food and beverage industry to influence food and nutrition policies

**DOI:** 10.1186/s12966-019-0900-8

**Published:** 2020-01-29

**Authors:** D. Vandenbrink, E. Pauzé, M. Potvin Kent

**Affiliations:** 1 Faculty of Medicine, University of Ottawa, 451 Smyth Rd, Ottawa, ON K1H 8M5 Canada; 2 School of Epidemiology and Public Health, Faculty of Medicine, University of Ottawa, 600 Peter Morand Crescent, Room 207F, Ottawa, Ontario K1G 5Z3 Canada; 3 School of Epidemiology and Public Health, Faculty of Medicine, University of Ottawa, 600 Peter Morand Crescent, Room 301J, Ottawa, Ontario K1G 5Z3 Canada

**Keywords:** Nutrition policy, Lobbying, Industry, Public health, Obesity prevention, Political activity

## Abstract

**Background:**

Unhealthy food environments contribute to the rising rates of obesity and diet-related diseases. To improve the Canadian nutritional landscape, Health Canada launched the *Healthy Eating Strategy* in October 2016 which involved several initiatives including the restriction of unhealthy food marketing to children, the reduction of sodium in the food supply and the introduction of front-of-package labelling. Subsequently, various stakeholders engaged in discussions with Health Canada. This study sought to describe the interactions between Health Canada and industry and non-industry stakeholders and to identify the strategies used by industry to influence food and nutrition policy in Canada.

**Methods:**

Documents such as correspondences and presentations exchanged in interactions between Health Canada and stakeholders regarding the *Healthy Eating Strategy* were obtained from Health Canada’s Openness and Transparency website. The participating stakeholders of each interaction and the topics discussed were determined and described quantitatively. A directed content analysis was then conducted to identify the strategies employed by industry to influence policy. This was guided by a previously developed coding framework that was adapted during analysis.

**Results:**

A total of 208 interactions concerning the *Healthy Eating Strategy* occurred between October 2016 and June 2018. Of the interactions for which documents were received (*n* = 202), 56% involved industry stakeholders, 42% involved non-industry stakeholders and 2% involved both. Industry stakeholders were more likely to initiate interactions with Health Canada (94% of their interactions) than non-industry stakeholders (49%). Front-of-package labelling was the most frequently discussed topic by industry stakeholders (discussed in 49% interactions involving industry) while non-industry stakeholders most frequently discussed the *Healthy Eating Strategy* as a whole (discussed in 37% of interactions involving non-industry). A wide variety of strategies were used by industry in their attempts to influence policy. Those most frequently identified included: “framing the debate on diet- and public health-related issues”, “promoting deregulation”, “shaping the evidence base”, “stressing the economic importance of industry”, and “developing and promoting alternatives to proposed policies”.

**Conclusion:**

Industry stakeholders are highly active in their attempts to influence Canadian nutritional policies. Policymakers and public health advocates should be aware of these strategies so that balanced and effective food and nutrition policies can be developed.

## Background

Between 1975 and 2016, global obesity rates nearly tripled, rising to approximately 13% of the global adult population [1]. In Canada, 64% of adults over the age of 18 had excess weight or obesity in 2017 [2]. Obesity is clearly associated with increased all-cause mortality and is a major risk factor for many non-communicable diseases (NCDs), including Type 2 diabetes, hypertension, obstructive sleep apnea, coronary artery disease and certain cancers [2–5]. Obesity also results in significant costs for national economies and healthcare systems; in 2006, the direct healthcare cost attributable to overweight and obesity in Canada was six billion dollars [6].

Although the dramatic rise in obesity rates is a complex and multi-faceted issue, changes to the global food system appear to be an important driver, as increases in global obesity rates correspond with greater accessibility to food that is affordable, highly processed and effectively marketed [7, 8]. In Canada, changes to the food environment have caused the average percentage of calories obtained from ultra-processed foods to rise from 28.7 to 61.7% between 1938 and 2011 [9]. Consequently, in an effort to improve the Canadian food environment and thereby stem the tide of increasing rates of obesity and diet-related NCDs, Health Canada launched the *Healthy Eating Strategy* in October of 2016 [10]. The *Healthy Eating Strategy* consists of six major components: the revision of Canada’s Food Guide, the restriction of unhealthy food marketing to children, the introduction of front-of-pack labelling on food products, the reduction of sodium in food products, the elimination of industrially produced trans fat from the Canadian food supply, and the implementation of strategies to improve the affordability and accessibility of nutritious food in northern communities. In the spirit of transparency, Health Canada instituted a policy which regulates its communication with stakeholders regarding the *Healthy Eating Strategy* [11]. Under this policy, any communication between Health Canada and stakeholders which is intended to inform policy development related to the *Healthy Eating Strategy*, excluding written submissions to formal consultations, is published on the Health Canada website [12].

Previous research has shown that the food and beverage industry is an important player in shaping food environments and often interacts with public agencies to influence nutrition policies [13–15]. To inform policy development and protect their commercial interests, food and beverage industry groups utilize many strategies such as actively lobbying governments [16, 17], vilifying scientific evidence that does not support their position [18], funding research studies designed to support their position [19], asserting that obesity is a matter of personal responsibility [20], as well as many other tactics [21]. Consequently, the influence of industry has been cited as a barrier to nutrition policy change [13].

Recent efforts to monitor and characterize the strategies utilized by industry to influence nutrition policy development have been undertaken in France, Australia and Fiji [16, 17, 22, 23]. However, to our knowledge, no equivalent studies have been performed in Canada. Furthermore, Health Canada’s Openness and Transparency policy offers a unique opportunity to identify the strategies utilized by industry to shape nutrition policy. Therefore, our research study sought to answer the following questions: 1) What is the frequency and nature of interactions between Health Canada and industry stakeholders and how does it compare with that of non-industry stakeholders? and 2) What strategies and practices are being used by industry stakeholders in an attempt to influence Health Canada with respect to the *Healthy Eating Strategy*?

## Methods

To answer these questions, the documents available through Health Canada’s Openness and Transparency website were obtained, categorized and qualitatively analyzed. For each interaction with Health Canada, this website lists the name(s) of participating stakeholder(s), the date of the interaction and the topic of discussion, while the documents shared in the interaction are available upon request. All documents listed on Health Canada’s “Meetings and correspondence on healthy eating” website were requested in June–July of 2018, using the website’s online order forms [12]. These documents originated from interactions—either meetings or letter/email correspondences—between Health Canada and stakeholders from October 2016 to June 2018. Documents added to the website after July 17, 2018 were neither requested nor included in the analysis.

### Identifying stakeholders involved in each interaction

Since Health Canada’s website did not always report all stakeholders involved in the listed interactions, documents associated with each interaction were reviewed and all participating stakeholders were recorded. In certain instances, many stakeholders were involved in a single interaction with Health Canada. In some of these cases, the documents did not list all the stakeholders present or simply noted that “various participants” were present (*n* = 11); if this occurred, stakeholders were not recorded. However, in some cases, a very large number of participating stakeholders were listed, and it was not feasible to record each individual stakeholder. Therefore, if all participating stakeholders were listed and the interaction involved 10 stakeholders or less (*n* = 183), each stakeholder was recorded. If the interaction involved more than 10 stakeholders (*n* = 8), these participants were not recorded.

### Identifying stakeholders responsible for analyzed documents

Each document analyzed in this study was also attributed to specific stakeholders. These documents included letters and emails from stakeholders to Health Canada, notes from meetings, and documents summarizing stakeholder feedback. In some instances, stakeholders shared documents with Health Canada which were authored by another individual or organization (*n* = 9). In these cases, the document was attributed to the stakeholder that shared the material, rather than to the author. On one occasion, a public relations firm contacted Health Canada on behalf of a food company; this letter was attributed to both organizations. Additionally, when multiple stakeholders were present in a meeting, any documents shared in the meeting were assumed to be shared by all parties. An exception to this rule was made if individual organizations made presentations to Health Canada as part of a multi-stakeholder meeting; in this circumstance, the document containing the presentation was only attributed to the presenting organization.

### Classifying stakeholders

Each identified stakeholder was classified as an industry or non-industry stakeholder. Industry stakeholders included any organization which could conceivably have a commercial interest, such as food and beverage companies and associations, advertising companies and associations, professional sports leagues, and public relations or law firms representing industry. Non-industry stakeholders included organizations which have no commercial interest, including public health organizations, health advocacy groups and non-profit community organizations. Four organizations had representation from both industry and non-industry (i.e. members from academia, government and the private sector). These organizations were classified as industry stakeholders as at least some of their members had commercial interests related to the Healthy Eating Strategy. Each organization’s website was assessed to facilitate their classification. Industry stakeholders were further classified according to the organization’s specific industry, such as food, advertising or sports. Organizations which acted on behalf of other industry groups, such as public relations and law firms, were classified as “unspecified.”

### Classifying topics discussed in interactions with Health Canada

Documents associated with each interaction were read by the first author (D.V.) and classified according to the following topics: Canada’s Food Guide, front-of-package labelling, Healthy Eating Strategy (if the entire strategy was discussed in a general sense), marketing to children, nutrition labelling (if the Nutrition Facts Table or labelling located at the back of products was discussed), sodium, sugar, and dietary fat (including trans and saturated fat). If multiple topics were identified within the same interaction, all of these were recorded. The topic of “sugar” is not a specific component of the *Healthy Eating Strategy* but was the primary focus of some discussions and, therefore, was listed as a unique topic.

### Classifying interactions and documents by stakeholder

Once the stakeholders were classified, each individual interaction was also classified as involving “industry”, “non-industry” or both “industry and non-industry”. For interactions classified as “industry”, the interaction was further characterized according to the specific field of industry represented: food, advertising, sports or unspecified. “Unspecified” was defined as interactions in which a public relations or law firm contacted Health Canada, presumably on behalf of an industry organization, but that organization was not specified. However, if a public relations or law firm contacted or met with Health Canada on behalf of an industry organization that was identified, the interaction was classified according to the specific industry of the represented stakeholder (*n* = 9). If multiple industry groups from different fields were present at a meeting or involved in a correspondence (*n* = 2), the interaction was classified according to the industry with the most representation. Finally, each individual document was classified in a similar manner.

### Additional characteristics of the interactions

Health Canada’s website describes whether interactions were initiated by the stakeholder or by Health Canada, and if the interaction was a meeting or correspondence. This information was recorded.

### Qualitative analysis: identifying strategies used by industry stakeholders

A qualitative document analysis was performed to identify the strategies used by industry stakeholders to influence Health Canada. Throughout the analysis, we sought to follow the accepted standards for reporting qualitative research, as outlined by O’Brien et al. [24]. In this study, a positivist research paradigm was adopted and directed content analysis was used to identify industry strategies [25]. Directed content analysis was chosen because we sought to build upon pre-existing theory in the field of corporate political activity by using an a priori framework developed by Mialon et al. to facilitate the document analysis [21]. Mialon et al. developed this framework following a review of the literature; it is designed to categorize the political activities of food companies into various strategies, practices and mechanisms. The six broad strategies that make-up this framework are described in Table 1. Mialon et al.’s framework is organized such that various “mechanisms” are nested within categories of “practices”, which, in turn, are nested within broader “strategies”. Therefore, during the coding process, passages were coded line-by-line at the lowest subcategory; consequently, if a passage was coded to the level of “mechanism”, it was then automatically coded to the corresponding “practice” and “strategy”. Following an initial familiarization period, the coding was conducted in an iterative manner, using *N Vivo 12* software, with several passes through the documents to recheck the coded passages.

**Table 1:** Strategies used by food industry stakeholders in their political activities, as defined by Mialon et al. [16]

Strategies	Description
Constituency building	Activities whose goals are to gain favor among the public and other important stakeholders like the media and public health community (e.g. getting involved in the community, establishing relationships with health organizations)
Financial incentive	The use of financial incentives to influence decision-makers (e.g. providing funds or giving gifts to politicians)
Information and messaging	Dissemination and framing of information in manners that favor the food industry’s activities and interests (e.g. shaping the evidence base by disseminating non-peer reviewed research, shifting blame away from industry, stressing their economic importance)
Legal	Use of legal means to oppose or influence policies (e.g. threaten legal action against critics or public policies)
Opposition, destabilisation and fragmentation	Destabilisation of groups or individuals whose opposition to the food industry’s products or practices may undermine their interests (e.g. criticize community organizations and advocates)
Policy substitution	Proposing alternatives to regulatory action (e.g. industry self-regulation)

During the iterative coding process, the framework was modified to facilitate accurate classification of the data, as novel strategies were identified, and other categories were determined to be irrelevant to our study. The first author kept a running journal during the coding process, in which he identified ambiguities and tracked strategies which emerged from the documents. Some significant changes from the Mialon et al. framework [21] are worth noting. For instance, the practice of “lobbying policymakers” and “establishing relationships with policymakers” and their associated subcategories were removed from the framework because every interaction between industry stakeholders and Health Canada was deemed an instance of lobbying and an attempt to build relationships with policymakers. The mechanism of “cherry picking data that favours the industry” was also removed because this activity was difficult to establish objectively. Minor changes were made to mechanism descriptions to more accurately represent our dataset. The study’s final coding framework, with detailed descriptions of each code, is provided in the Additional file 1.

Finally, in addition to quantitatively describing the occurrence of each strategy, practice and mechanism, a qualitative description of these was formulated and illustrative passages were chosen to provide an explanatory account of the strategies used by industry to influence Heath Canada.

The qualitative analysis was performed by the first author (D.V.), a medical student with research experience in quantitative methods but limited prior familiarity with qualitative research methods and the research topic. Prior to beginning this analysis, he conducted a literature review on the political activities of the food industry and spent a significant amount of time learning about qualitative research methods. Following the framework analysis performed by D.V., the framework and all coded passages were revised by the second author (E.P.), a registered dietitian with experience in qualitative coding and expertise in food industry practices, including their marketing practices and political activities. Disagreements were resolved through consensus. The entire work was guided by an applied public health researcher with extensive expertise in mixed methods research, particularly in the field of food and beverage marketing to children, food industry self-regulation and the political activities of the food sector (M.P.K.).

### Evaluation of citations made by industry stakeholders

During the framework analysis process, citations used by industry to support their arguments were identified. This cited evidence was sought by using Google, Google Scholar and the university library services. When the evidence was found, the article or report was evaluated to determine if the study was published, if it was peer-reviewed and how it was funded. In addition, if a document referred to research conducted by an external organization, this organization’s website was examined to determine whether it was funded by industry or a front group (i.e. a third-party organization that serves the interest of industry but whose relationship or sponsorship by industry is not or rarely disclosed).

### Quantitative analysis

Descriptive statistics were performed using *IBM SPSS Statistics* version 24 to describe Health Canada’s interactions with industry and non-industry stakeholders, including the frequency and nature of interactions, the topics of discussion, and the citations and strategies used by industry stakeholders. In interactions involving both industry and non-industry stakeholders (*n* = 4), discussed topics were attributed to both stakeholder categories. Documents associated with both industry and non-industry stakeholders (*n* = 5) were similarly treated. The proportion of interactions initiated by industry and non-industry was compared using a chi-square test. In this instance, interactions involving both industry and non-industry (*n* = 4) were excluded from the sample. A *p*-value lower than 0.05 was deemed statistically significant.

## Results

### Interactions between stakeholders and Health Canada

A total of 208 unique interactions concerning the *Healthy Eating Strategy* took place between Health Canada and stakeholders from October 2016 to June 2018. Because documents were not received for 6 interactions, only 202 interactions were included in the study (Table 2). Of these interactions, 56% (*n* = 113) involved industry stakeholders, 42% (*n* = 85) involved non-industry stakeholders and 2% (*n* = 4) involved both industry and non-industry stakeholders. Industry stakeholders were much more likely to initiate interactions with Health Canada than non-industry stakeholders; 94% (*n* = 106) of industry interactions were stakeholder-initiated, while 49% (*n* = 85) of non-industry interactions were stakeholder-initiated (X^2^ (1, *N* = 198) = 50.6, *p* < .000). In 6% of its interactions (*n* = 7), industry utilized an external lobbyist to assist in the discussion with Health Canada (data not shown).

**Table 2:** Description of interactions with Health Canada regarding the *Healthy Eating Strategy* by industry and non-industry stakeholders, October 2016 to June 2018

	Industry (*N* = 113)	Non-industry (*N* = 85)	Industry and non-industry (*N* = 4)	All interactions (*N* = 202)
	n (%)	n (%)	n (%)	n (%)
*Initiation of the interaction*				
Stakeholder initiated	106 (93.8)	42 (49.4)	2 (50)	150 (74.3)
Health Canada initiated	7 (6.2)	43 (50.6)	2 (50)	52 (25.7)
*Type of interaction*				
Correspondence	33 (29.2)	13 (15.3)	2 (50)	48 (23.8)
Meeting	80 (70.8)	72 (84.7)	2 (50)	154 (76.2)

As shown in Table 3, front-of-package labelling was the most discussed topic by industry stakeholders with Health Canada (discussed in 48.7% of interactions; *n* = 57). By contrast, front-of-package labelling was discussed in only 27% of interactions (*n* = 24) involving non-industry stakeholders. Marketing to kids was also more commonly discussed by industry (13.7% of interactions; *n* = 16) than by non-industry (7.9% of interactions; *n* = 7), as was sodium (12.0% of industry interactions; *n* = 14 vs. 4.5% of non-industry interactions; *n* = 4). On the other hand, the Canada Food Guide was more commonly discussed by non-industry (31.4% of interactions; *n* = 28) than by industry (12.0% of interactions; *n* = 14).

**Table 3:** Number and frequency of the topics discussed during interactions between Health Canada and stakeholders regarding the *Healthy Eating Strategy*, October 2016 to June 2018

Topic	Industry n (%)^a^	Non-Industry n (%)^a^	All interactions n (%)^a^
Canada Food Guide	14 (12.0)	28 (31.4)	42 (20.8)
Front-of-package labelling	57 (48.7)	24 (27.0)	79 (39.1)
Healthy Eating Strategy	43 (36.8)	33 (37.1)	74 (36.6)
Marketing to children	16 (13.7)	7 (7.9)	23 (11.4)
Nutrition labelling	6 (5.1)	5 (5.6)	11 (5.4)
Sodium	14 (12.0)	4 (4.5)	18 (8.9)
Sugar	0 (0)	5 (5.6)	5 (2.5)
Dietary fat	4 (3.4)	4 (4.5)	8 (4.0)
Total number of interactions^b^	117	89	202

^a^Percentage of interactions in which the topic was discussed. The total number of topics exceeds the number of interactions between Health Canada and stakeholders because multiple topics were often discussed in a single interaction

^b^The sum of interactions involving industry (*n* = 117) and non-industry (*n* = 89) stakeholders exceeds the total number of interactions (*N* = 202) because four interactions involved both type of stakeholders. Topics discussed in these mixed interactions were attributed to both industry and non-industry stakeholders

Of the 801 documents requested from Health Canada, 94% were received before the drafting of this article (*n* = 753). Documents originating from Health Canada containing no information from stakeholders (e.g. a letter sent by Health Canada that received no reply or presentations given by Health Canada) were excluded from the study (*n* = 278). As such, a total of 475 stakeholder documents were reviewed. As shown in Table 4, industry stakeholders were associated with 59.6% of the documents (*n* = 271), with most of these (94.8%; *n* = 257) coming from interactions involving food industry stakeholders. Overall, 127 different organizations participated in discussions with Health Canada; 52.8% of these were industry stakeholders (*n* = 67; Additional file 1: Table S1).

**Table 4:** Number and percentage of documents resulting from interactions between Health Canada and industry and non-industry stakeholders regarding the Healthy Eating Strategy, June 2016 to October 2018 (*N* = 475)

Type of Stakeholder	n (%)^a^
Industry	283 (59.6)
Advertising	18 (6.6)
Food	257 (94.8)
Sports	2 (0.7)
Unspecified	6 (2.2)
Non-Industry	197 (41.5)

^a^The sum of documents classified as involving industry and non-industry stakeholders exceeds the total number of documents because five documents were attributed to both industry and non-industry stakeholders

### Industry strategies

A wide variety of strategies were utilized by industry stakeholders in their attempts to influence Health Canada’s development of nutrition policy. As shown in Table 5, a total of six strategies, ten practices and nineteen mechanisms were identified.

**Table 5:** Number of interactions in which strategies, practices and mechanisms† were used by industry to influence Health Canada regarding the *Healthy Eating Strategy*, October 2016 to June 2018 (*n* = 113)

Strategies	Practices	Mechanisms	Number of times used (% of interactions in which the tactic was used)	Number of stakeholders which used the tactic
Constituency building			3 (2.6)	4
	Establish relationships with key opinion leaders and health organizations	Promote interactions with health-related organizations	1 (0.9)	2
	Seek involvement in the community		3 (2.6)	2
		Support physical activity initiatives	3 (2.6)	2
		Undertake corporate philanthropy	3 (2.6)	2
Financial incentive	Fund government initiatives		1 (0.9)	1
Information and messaging			57 (48.7)	44
	Frame the debate on diet- and public health-related issues		48 (41.0)	39
		Emphasize industry’s actions to address obesity and chronic disease	24 (20.5)	26
		Highlight beneficial actions and initiatives unrelated to obesity and chronic disease	7 (6.0)	9
		Promote the good intentions and stress the good traits of industry and industry products	33 (28.2)	29
		Shift the blame and draw attention away from industry, e.g. focus on individual responsibility, role of parents, physical inactivity	24 (20.5)	21
		Take quotations out of context to support industry positions	1 (0.9)	2
	Promote deregulation		33 (28.2)	21
		Demonize the nanny state	2 (1.7)	4
		Emphasize the paucity of evidence in support of proposed initiatives	8 (6.8)	8
		Highlight the potential burden, challenges and unintended consequences associated with regulation (losses of jobs, administrative burden, worse public health outcomes)	30 (25.6)	18
	Shape the evidence base on diet- and public health-related issues		31 (26.5)	31
		Criticize the evidence and assert that studies are junk science	3 (2.6)	2
		Disseminate and use non-peer reviewed or unpublished evidence	13 (11.1)	16
		Emphasize the complexity and uncertainty in science	6 (5.1)	6
		Fund research, including through academics, ghost writers, own research institutions and front groups	18 (15.4)	15
		Make general references to supporting evidence without providing specific citations	9 (7.7)	7
		Pay scientists and health professionals as advisors, consultants or spokespersons	8 (6.8)	5
		Provide industry-sponsored education materials	5 (4.4)	9
		Demonstrate reluctance to provide information that is not publicly available	6 (5.1)	7
	Stress the economic importance of the industry, including the number of jobs supported and the money generated for the economy		22 (18.8)	27
Legal	Influence the development of trade and investment agreements to include clauses favourable to industry		5 (4.4)	10
Opposition fragmentation and destabilization	Criticize governmental and community organizations and advocates		3 (2.6)	3
Policy substitution	Develop and promote alternatives to proposed policies including revised policies, voluntary codes, self-regulation and non-regulatory initiatives		40 (34.2)	23
Total Number of Interactions between Industry and Health Canada			117	

^†^Based on an adapted framework developed by Mialon et al. [21]

#### Information and messaging

“Information and messaging” was the strategy most commonly used by industry; it was identified in 48.7% (*n* = 57) of their interactions with Health Canada. In 41.0% (*n* = 48) of these interactions, industry was observed to use the practice of “framing the debate on diet- and public health-related issues”. For example, industry stakeholders commonly used the mechanism of “emphasizing industry’s actions to address obesity and chronic disease” (20.5% of interactions; *n* = 24), such as when one industry association stated in a letter that “our members responded positively to the voluntary initiative to reduce/eliminate trans fat as demonstrated through research and development … Similarly [our] members have made sodium reduction a priority and reduced sodium in pantry bread by 11%.”

Industry stakeholders also frequently employed the mechanism of “promoting the good intentions and stressing the good traits of industry and industry products” (28.2% of industry interactions; *n* = 33). For instance, a large food company stated in a letter: “… we want to contribute our knowledge and resources to help the government develop and promote a modernized, impactful Canada Food Guide.” Similarly, an industry association promoted the beneficial qualities of their product in a document stating: “For as long as there have been Canadian dairy farms, Canadian milk has been a trusted source of vitamins, minerals, and protein. Milk products have stood the test of time, and remain scientifically proven to be one of the truly nutritious and healthy foods in our diet.”

In interactions with Health Canada, industry used the mechanism of “shifting the blame and drawing attention away from industry, e.g. focusing on individual responsibility, role of parents, physical inactivity” (20.5% of interactions; *n* = 24). For instance, an email sent by the representative of a food company stated: “We support the efforts of Health Canada in this area as we are also very committed to providing easy to understand information to help consumers make healthier food choices.” In a similar manner, an industry association “drew attention” from their own goods to other food products: “For example, new labels on some nutrient-rich foods like cheese and flavoured yogurt that include warning labels could discourage their consumption altogether, despite their scientifically proven nutritional benefits. In contrast, things like potato chips and diet soda would not have “warning“ labels and could therefore be perceived as more “healthy“ than nutrient-rich foods”.

Industry stakeholders also sought to “highlight beneficial actions and initiatives unrelated to obesity and chronic disease” including food safety, environmental sustainability and animal welfare (6% of interaction; *n* = 7). For instance, a brochure shared by one company touted their responsible corporate practices stating: “By sourcing responsibly, protecting our environment and making a positive difference in our communities we strive to be an exemplary corporate citizen and use our scale to tackle some of society’s toughest challenges.” Concern for food safety and security was also expressed by a food production group: “Fruit and vegetable growers rely on a wide range of crop protection strategies, such as integrated pest management plans and government-approved pesticides, to ensure the health of crops, the safety of Canadians, and Canada’s overall food security.”

Another frequent practice used by industry was to “promote deregulation” (28.2% of interactions; *n* = 33). Industry regularly employed the mechanism of “highlighting the potential burden, challenges and unintended consequences associated with regulation” (25.6% of interactions; *n* = 30). For instance, a letter from several food associations stated: “As has already been documented and shared with government officials, [an] economic analysis demonstrated that the potential restrictions on marketing to children floated by Health Canada last summer would have multi-billion-dollar impacts on the Canadian economy including more than $7 billion in lost GDP, 30,000 plus lost jobs and a reduction in annual ad spend of more than $1.1 billion, including more than $300 million in reduced spend with Canada’s struggling broadcast industry.” Industry also argued that Health Canada’s proposed regulations would have detrimental effects on public health, rather than the intended positive impact. In a presentation, an association argued that “removing 100% juice from [Canada’s Food Guide] will negatively impact Canadians’ health.” Industry also promoted deregulation through the mechanism of “emphasizing the paucity of evidence in support of proposed initiatives” (6.8% of interactions; *n* = 8), such as stated in a presentation given by an association: “With no clear evidence that the policy intervention will yield positive health outcomes, we need to take the time to get this right”.

Industry frequently employed the strategy of “shaping the evidence base on diet- and public health-related issues” (26.5% of interactions; *n* = 31) through a variety of mechanisms. For instance, industry commonly provided citations in support of their arguments; however, out of 75 identified citations, only 54 were found online and none stated as being “available upon request” were received, when requested by the authors (Table 6). Of the identified citations, 9% were unpublished (*n* = 5), 35% were not peer-reviewed (*n* = 19) and 43% were either fully or partially funded by industry (*n* = 23). As an example, one food association provided Health Canada with results from a survey on Canadians’ attitudes toward food and beverage labelling that its organization had commissioned from an external polling and market research company. Findings from this research, which had not been formally published, suggested, for example, that the majority (60–66%) of surveyed Canadians thought that information about levels of sodium, sugar and saturated fat “was available and clear enough” on current food labels.

**Table 6:** Evaluation of supporting citations found in documents provided by industry in discussions with Health Canada regarding the *Healthy Eating Strategy*, October 2016 to June 2018

	n (%)
*Citations mentioned in documents provided by industry (n = 75)*	
Citations found	54 (72.0)
Citations not found	21 (28.0)
*Characteristics of citations that were found (n = 54)*	
Published	
Yes	49 (90.7)
No	5 (9.3)
Type of Publication	
Government publication	6 (11.1)
Non-peer reviewed article or report	19 (35.2)
Peer-reviewed article	29 (53.7)
Funding	
Industry	23 (42.6)
Not industry	24 (44.4)
Unknown	7 (13.0)

In some instances, industry stakeholders used the mechanism of “demonstrating reluctance to share information” (5% of interactions; *n* = 6). For example, in a joint letter from various food and advertising associations, a cost-benefit analysis (CBA) survey sent by Health Canada to industry stakeholders to assess the impact of proposed food marketing restrictions was criticized for being “so ambiguous that any responses provided will be impossible to interpret”, among other reasons. Their criticism of the survey was supported by an assessment conducted by an external consulting firm with expertise in regulatory assessments. Regarding survey questions pertaining to the resources and methods employed by food companies to market to children, the consultant stated that Health Canada did “not require such information at a company-level to prepare cost estimates for a CBA” and referred to an impact assessment prepared by an advertising association.

Industry stakeholders also utilized the mechanism of “making general references to supporting evidence without providing specific citations” (7.7% of interactions; *n* = 9). For instance, a letter from a food production group states that “Dairy and meat products have long been considered an integral part of a healthy, balanced diet, and the evidence supporting this hasn’t changed”, but no specific evidence is provided. It was also found that industry employed the mechanisms of “criticizing the evidence and asserting that studies are junk science” (2.6% of interactions; *n* = 3), “paying scientists or health professionals as advisors, consultants or spokespersons” (6.8% of interactions; *n* = 8), and “providing industry-sponsored education materials” (4.4% of interactions; *n* = 5).

In discussions with Health Canada, industry stakeholders regularly used the practice of “stressing the economic importance of the industry, including the number of jobs supported and the money generated for the economy” (18.8% of interactions; *n* = 22). In a brochure provided to Health Canada, one company states that “Through a network of corporate and independent businesses, [they] service more than 17 million Canadians every week. With more than 2,400 corporate, franchised and associate-owned locations, [the company], its franchisees and Associate-owners employ approximately 200,000 full and part-time employees making it one of Canada’s largest private sector employers.” Similarly, two food production groups state in a joint letter, “As you are fully aware, agriculture and agri-food is Canada’s top job creator” while an association declared in a presentation that “Retail is Canada’s largest employer - over 2.1 million Canadians.”

#### Policy substitution

Industry stakeholders frequently employed the practice of “developing and promoting alternatives to proposed policies including revised policies, voluntary codes, self-regulation and non-regulatory initiatives” in discussions with Health Canada. In fact, this practice was identified in 34.2% of industry interactions (*n* = 40). This practice is evident in a letter sent by an association stating they “were the first in the food and beverage sector to voluntarily restrict the sale of beverages in elementary, middle, and high schools. [The association] also has an additional marketing code that specifically prohibits the marketing of caffeinated energy drinks to children.” Industry stakeholders would also highlight the success of their voluntary initiatives, as described in an infographic provided by an association. “Current data … indicates, that since our 2016 baseline report, there has been at least a 9% drop in calories consumed per capita from non-alcoholic beverages in Canada since 2015. [The initiative] is working.” Moreover, notes from a meeting with an association on front-of-package labelling indicates that industry proposed alternatives to initiatives being considered by Health Canada: “[Our association] representatives indicated that the “Facts up Front“ approach they are proposing would be more meaningful for Canadians consumers as it complements the Nutrition Facts table (NFt), is simpler and is non-discriminatory (would be present on all food products).” At times, rather than presenting alternatives, industry groups lobbied for revisions to proposed policies. For instance, notes from a meeting with one food company indicate that “[the company] presented their progress on sodium reduction and food categories where there have been challenges meeting the sodium reduction benchmark levels. [The company] proposed ideas for how to revise the sodium reduction benchmark levels.”

#### Other strategies

Additional strategies utilized by industry were identified less frequently. The strategy of “constituency building” was identified in 2.6% of industry interactions (*n* = 3). One instance of the practice of “establishing relationships with key opinion leaders and health organizations” was identified. As expressed in a letter, an association developed a proposal for front-of-package labelling in consultation with a non-governmental health organization. Additionally, one example of the practice of using a “financial incentive” was seen in a letter from a food company, when they referred to their “significant contributions of cash and in-kind support to … the Nutrition Facts Education Campaign in partnership with Health Canada.”

The use of the “legal” strategy was observed in 4.4% of interactions (*n* = 5), as industry groups sought to influence the development of trade and investment agreements. For example, in a brochure provided to Health Canada, an association stated, “Promoting trade and investment with export markets is a priority for the Canadian produce sector … Canada’s fruit and vegetable industry urges the federal government to … Increase cooperation and alignment on plant health, customs and recognition of food safety systems, as increased cooperation will facilitate trade and provide benefits to industry and consumers, while protecting Canada’s food security, people and environment.”

The practice of “opposition, fragmentation and destabilization” was observed in 2.6% of interactions (*n* = 3). Three situations were identified in which industry used the practice of “criticizing governmental and community organizations and advocates”. For instance, a food association wrote in a letter, about an earlier multi-stakeholder meeting: “that meeting was an excellent demonstration that Health Canada has lost its way on the obesity issue”.

## Discussion

This study clearly demonstrates that the Canadian food and beverage industry is actively involved in direct lobbying of policymakers/civil servants working on various food and nutrition policies. Industry stakeholders appear to be very concerned about the implications of the *Healthy Eating Strategy,* given that collectively, they have been involved in 117 interactions with Health Canada between October 2016 and June 2018 and have initiated more than 90% of these interactions.

To our knowledge, this is the first study evaluating the lobbying strategies used by industry to influence Canadian food and nutrition policies. Our study found that Canadian food industry utilized similar strategies to those used by their counterparts in other jurisdictions [16, 17, 22, 23] and by other industries such as alcohol and tobacco [20, 26–28]. For instance, Canadian industry stakeholders commonly used the practice of “framing the debate on diet- and public health-related issues”, through the mechanisms of “emphasizing industry’s actions to address obesity and chronic disease, “promoting the good intentions and stressing the good traits of industry” and “shifting the blame and drawing attention away from industry”. These strategies were also identified from publicly available information in Australia and France [17, 22]. Similarly, previous research has shown that the food industry has long been known to shape the evidence base on diet and public health-related issues [14, 19]. This practice was frequently observed in the current study, as passages in industry documents were identified as using the mechanisms of “funding research”, “disseminating and using non-peer reviewed or unpublished evidence”, “paying scientists or health professionals as advisors, consultants or spokespersons” and “criticizing the evidence” supporting Health Canada’s proposals. However, analysis of the material cited by industry indicated that a significant proportion of the evidence was either unpublished, non-peer reviewed, or industry funded.

In an analysis of lobbying submissions from the Canadian food industry in response to trade agreement negotiations, Friel et al. [29] found that the Canadian food industry often stressed their economic importance. This finding was also evident in our study. Industry stakeholders frequently employed the practice of “stressing the economic importance of the industry, including the number of jobs supported and the money generated for the economy”. Furthermore, when addressing specific proposals, industry often used the practice of “promoting deregulation” through the mechanism of “highlighting the potential burden, challenges and unintended consequences associated with regulation”. Extensive promotion of deregulation and highlighting the burden associated with nutrition policies has also been utilized by the American, Australian and Fijian food industries [16, 23, 30].

Although industry organizations were eager to highlight the successes of their voluntary or self-regulatory initiatives, independent evaluations of these programs are less positive [31–39]. For example, an association promoted a front-of-packing labelling scheme developed by the food industry which mirrors the nutrition information available in the Nutrition Facts table (i.e. guidelines on daily recommended intakes) [40]. Scientific evidence, however, has shown that other more interpretative labelling schemes are better at improving consumers’ understanding of nutrition information and their food choices [32–34]. Similarly, various food and advertising industry stakeholders underscored their voluntary restrictions of marketing to children and, specifically, their participation in the Canadian Children’s Food and Beverage Advertising Initiative (CAI). However, several studies have shown that the CAI has been ineffective at improving the healthfulness of foods and beverages promoted to children on television and the internet [35–39].

Because the current study only analyzed documents submitted to Health Canada in response to the *Healthy Eating Strategy*, some strategies commonly used by the food industry, such as “seeking involvement in the community” [17, 22, 23], were rarely identified in their correspondences with Health Canada. This might be the case as such initiatives may serve as public relations or marketing strategies aimed at gaining favor or fostering positive sentiments among the public rather than influencing policymakers. Other research has in fact shown that the Canadian food industry is also heavily involved in corporate social responsibility activities related to nutrition and physical activity, including many directed at children (Potvin Kent, unpublished).

A valuable secondary outcome of this study was the evaluation of the utility of Mialon et al.’s framework in the analysis of a novel dataset [21]. In general, the framework was highly applicable to the documents obtained from Health Canada and greatly facilitated our analysis. However, because Mialon et al.’s framework was created to analyze information from a wide range of sources, including industry websites, news releases, and even social media accounts, the framework required modification for this study. For instance, the practices of “lobbying policymakers” and “establishing relationships with key opinion leaders and health organizations”, as described in Mialon et al.’s framework, were not applicable to this dataset because every interaction between industry and Health Canada was an example of these practices. Moreover, this study identified novel mechanisms which were not included in the Mialon et al.’s framework. For instance, to “shape the evidence base on diet and health-related issues”, industry stakeholders were found to use the mechanisms of “making general references to supporting evidence without providing specific citations” and “demonstrating reluctance to share information”. Industry stakeholders also used the mechanism of “emphasizing the paucity of evidence in support of proposed initiatives” to further the strategy of “promoting deregulation”. While these mechanisms were not identified by Mialon et al., they did fit within the broader corporate strategies and practices identified in their framework. As such, Mialon et al.’s framework is highly useful in facilitating analysis of documents and other data sources when categorizing the food industry’s corporate political activities however it must be adapted to the specific dataset in question.

### Study limitations

The limitations of this research are important to highlight. As previously mentioned, this study only analyzed documents regarding the *Healthy Eating Strategy* that were submitted to Health Canada outside formal consultation processes. The analysis of documents submitted through formal consultations or other sources, such as company websites and media reports, may have revealed the use of additional strategies. Our study was also limited by the quality of the available documents. In many cases, notes from meetings between Health Canada and stakeholders were sparse and did not provide a detailed account of their discussions. Furthermore, the topics discussed, and the strategies identified were likely influenced by the timing of our analysis. For example, Health Canada initiated consultations on front-of-package labelling in the summer and fall of 2017, so many discussions at that time addressed that topic. If our analysis had extended past June 2018, other topics may have been discussed more frequently and new strategies may have been identified. This study also underestimates the amount of lobbying that occurred as it only examined interactions with Health Canada and did not include interactions with members of Parliament or other government departments [41, 42]. For instance, a memo obtained through Access to Information suggests that the government department of Agriculture and Agri-Food Canada lobbied Health Canada in favor of the meat and dairy industries’ interests when asked to provide input regarding the revisions to Canada’s Food Guide [41].

Finally, it is important to acknowledge that this document analysis cannot account for what was said and emphasized by industry stakeholders in meetings with Health Canada nor can it establish whether food industry lobbying influenced the development of food and nutrition policies and regulations. Interviews with key informants like civil servants, public health advocates, politicians and industry insiders could provide more information on industry strategies and their involvement in the policy-making process [16, 21]. Notwithstanding these limitations, this study provided valuable information about the strategies used by industry stakeholders in their attempt to influence food and nutrition policy in Canada. Future research on the political activities of the Canadian food and beverage industry should examine additional sources of information and other activities such as corporate social responsibility initiatives, participation in health-related working groups, sponsorship of professional bodies and their activities, and funding of scientific research or conferences [21].

## Conclusions

This study demonstrates that industry stakeholders are actively attempting to influence the development of food and nutrition policies and regulations in Canada. As such, industry must be recognized as a key determinant of the Canadian food environment and of Canadian public health. Though Health Canada’s transparency policy should be applauded, additional measures are likely needed to fully understand the scope of the food and beverage industry’s political activities and its influence on public health policies in Canada. To achieve balanced and substantively positive food and nutrition policy outcomes, policy-makers and public health advocates need to understand the role and strategies of industry.

## Supplementary information

**Additional file 1: **Coding Manual: Framework used to code documents provided by industry groups in discussions with Health Canada regarding the Healthy Eating Strategy. **Table S1.** Number and percentage of each type of stakeholder in contact with Health Canada regarding the *Healthy Eating Strategy*, October 2016 to June 2018.

## Data Availability

The documents analyzed in the current study are available upon request from Health Canada’s “Meetings and correspondence on healthy eating” website [https://www.canada.ca/en/services/health/campaigns/vision-healthy-canada/healthy-eating/meetings-correspondence.html].
