# Supplementary material for: Strategies used by the Canadian food and beverage industry to influence food and nutrition policies
Source: Int J Behav Nutr Phys Act. 2020 Jan 29;17:3. doi: 10.1186/s12966-019-0900-8 (PMC6988317; doi:10.1186/s12966-019-0900-8)
Supplement: Supplementary file 1 — Additional file 1: Coding Manual: Framework used to code documents provided by industry groups in discussions with Health Canada regarding the Healthy Eating Strategy. Table S1. Number and percentage of each type of stakeholder in contact with Health Canada regarding the Healthy Eating Strategy, October 2016 to June 2018. [file 12966_2019_900_MOESM1_ESM.docx]

**Additional file**

**Coding Manual: Framework used to code documents provided by industry groups in discussions with Health Canada regarding the Healthy Eating Strategy**

The following describes the framework which was utilized to code the documents shared by industry with Health Canada, in discussion surrounding the *Healthy Eating Strategy* between October 2016 and June 2018. The framework was adapted from Mialon et al. (1) and is designed to identify strategies used by industry to influence government for the purpose of protecting commercial interests.

The documents were coded at the lowest subcategory, which was either to the level of “Mechanisms” or “Practices”, depending on the topic under consideration. If a subcategory was identified as being utilized in a document, the supercategory was assumed to be utilized as well. In other words, if a passage was coded as a Mechanism in a document, the passage was automatically coded as the associated Practice and Strategy as well.

The framework is given below and the rationale for coding for each Mechanism is described. The rationale is intended to describe when a passaged was coded as a specific Mechanism.

| **Strategies** | **Practices** | **Mechanisms** | **Coding Rationale** |
| --- | --- | --- | --- |
| Constituency building |  |  |  |
|  | Establish relationships with key opinion leaders and health organizations |  |  |
|  |  | Establish informal relationships with key opinion leaders | Passages were coded if they described informal relationships between industry and key opinion leaders or if the passage indicated that such relationships exist |
|  |  | Promote interactions with health-related organizations | Passages were coded if they indicated that such relationships exist or if the passage expressed industry’s desire to establish such connections |
|  |  | Support professional organizations through funding and/or advertising in their publications | Passages were coded if they indicated that this had occurred |
|  | Establish relationships with the media to facilitate advocacy |  | Passages were coded if they indicated that this had occurred |
|  | Seek involvement in the community |  |  |
|  |  | Support events (such as for youth or the arts) and community-level initiatives | Passages were coded if they indicated that this had occurred |
|  |  | Support physical activity initiatives | Passages were coded if they indicated that this had occurred |
|  |  | Undertake corporate philanthropy | Passages were coded if they indicated that this had occurred |
| Financial Incentive |  |  |  |
|  | Fund and provide financial incentives to political parties and policymakers |  | Passages were coded if they indicated that this had occurred |
|  | Fund government regulatory initiatives |  | Passages were coded if they indicated that this had occurred |
| Information and Messaging |  |  |  |
|  | Frame the debate on diet- and public health-related issues |  |  |
|  |  | Demonstrate reluctance to share information | Passages were coded when industry refused to or expressed reluctance to provide information that was not publicly available |
|  |  | Emphasize industry's actions to address obesity and chronic disease | Passages were coded if they spoke of the industry’s *direct* role and *specific* actions in public health, including promoting healthy lifestyles, offering expertise and education, product reformulation/offering healthier products, giving consumers information to make informed choices, etc. Passages highlighting industry’s participation in consultations and revisions to proposed policies were NOT coded here; rather, these passages were coded under “Develop and promote alternatives to proposed policies including revised policies, voluntary codes, self-regulation and non-regulatory initiatives” |
|  |  | Highlight beneficial actions and initiatives unrelated to obesity and chronic disease | Passages were coded if they portrayed industry in a positive light by highlighting industry’s beneficial actions and initiatives unrelated to obesity and chronic disease (e.g. food safety, environmental sustainability, consumer rewards, animal welfare, etc) |
|  |  | Promote the good intentions and stress the good traits of industry and industry products | Passages were coded if they highlighted the good qualities of industry and industry’s products, or if they referred to good intentions of industry, such as expressions of desire and commitment to find solutions to the obesity and chronic disease problem |
|  |  | Shift the blame and draw attention away from industry, e.g. focus on individual responsibility, role of parents, physical inactivity | Passages were coded if they distracted from the role of industry and their products in contributing to the obesity problem. Distraction tactics include speaking of the importance of a balanced diet, arguing that all foods can be part of a healthy diet, highlighting the role of physical activity, emphasizing individual responsibility, touting the need for consumer education and improved nutrition literacy, stressing nuance and balance in nutrition messaging, and shifting blame onto other industry groups/products |
|  |  | Take quotations out of context to support industry positions | Passages were coded if they quoted scientists or articles to support the industry position, when the quotation, in context, was not actually intended to support that position. To determine if the quotation was intended to support the industry position, the quoted scientist was asked directly about their intention in providing the quotation |
|  | Promote deregulation |  |  |
|  |  | Demonize the nanny state | Passages were coded if they argued the government was being too heavy handed, too involved in the lives of the public or was taking free will away from individuals |
|  |  | Emphasize the paucity of evidence in support of proposed initiatives | Passages were coded if they stated that there was insufficient evidence to support Health Canada’s proposals, thereby implying that regulation is unwarranted |
|  |  | Highlight the potential burden, challenges and unintended consequences associated with regulation (losses of jobs, administrative burden, worse public health outcomes) | Passages were coded if they spoke of the economic cost, possible unintended adverse public health consequences, potential loss of investment, administrative challenges, or any other difficulty associated with regulation |
|  |  | Threaten to withdraw investments if new public health policies are introduced | Passages were coded if they constituted a direct threat to remove investment from Canada. If the passage only spoke of Canada becoming a less attractive market for investors, the passage was coded under “Highlight the potential burden and unintended consequences associated with regulation” |
|  | Shape the evidence base on diet- and public health-related issues |  |  |
|  |  | Criticize the evidence and assert that studies are junk science | Passages were coded if they criticized *studies* and/or *evidence.* By contrast, if passages criticized *scientists*, the passage was coded under “Criticize governmental and community organizations and advocates” |
|  |  | Disseminate and use non-peer reviewed or unpublished evidence | Passages were coded if they cited or referred to specific studies or data that are not published or are published in non-peer reviewed publications.  Specific citations were noted, tracked down and screened to determine if they were published in peer-reviewed journals. Passages referring to studies/data which were not found online were assumed to be unpublished data and, therefore, were also coded under this category. Also, passages referring to specific data when no citation was provided were assumed to be citing unpublished evidence and were coded under this category. However, passages were NOT coded under this node if they made general references to “evidence” or “science”; instead, these passages were coded under “Make general references to supporting evidence without providing specific citations” |
|  |  | Emphasize disagreement among scientists | Passages were coded if they specifically emphasized that *scientists* disagree about nutrition advice and/or scientific results. If passages stated that *data* or *evidence* is uncertain and conflicting, the passage was coded under “Emphasize the complexity and uncertainty in science” |
|  |  | Emphasize the complexity and uncertainty in science | Passages were coded if they highlighted the conflicting results in the scientific *evidence*. If a passage stated that *scientists* disagree about the evidence, the passage was coded under “Emphasize disagreement among scientists” |
|  |  | Fund research, including through academics, ghost writers, own research institutions and front groups | Passages were coded if they stated that the industry or company funds research, or if they referred to research studies that were identified as being industry funded. Searches for the citations were conducted and studies were evaluated to determine if they were funded by industry. References to research from external organizations were also found and evaluated to determine if the organizations were front groups for industry |
|  |  | Make general references to supporting evidence without providing specific citations | Passages were coded if they declared that “evidence” or “science” supports a certain position but no data was provided and no specific studies were cited |
|  |  | Participate in and host scientific events | Passages were coded if they indicated that this had occurred |
|  |  | Pay scientists or health professionals as advisors, consultants or spokespersons | Passages were coded if they directly referred to industry-supported or industry-employed scientists and health professionals (including dietitians). Passages were also coded if they indirectly indicated that a scientist was supported by or employed by industry (e.g. if a scientist contacted Health Canada as an employee of an industry organization) |
|  |  | Provide industry-sponsored education materials | Passages were coded if the passage was an actual educational document on healthy living or obesity prevention, which was sponsored or produced by industry. Passages were also coded if they described industry undertaking the practice of sponsoring and providing education material. Educational material which provided information about a company or industry, rather than about healthy living or obesity prevention, was NOT coded |
|  |  | Suppress or influence the dissemination of research | Passages were coded if they indicated that this had occurred |
|  | Stress the economic importance of the industry, including the number of jobs supported and the money generated for the economy |  | Passages were coded if they stated specific numbers of jobs supported, revenue generated, taxes paid, etc., by industry. Passages were also coded if they made general comments of the economic importance of a certain industry or company |
| Legal |  |  |  |
|  | Influence the development of trade and investment agreements to include clauses favourable to industry |  | Passages were coded if they stated industry’s concerns and desires with respect to trade agreements |
|  | Use legal action (or the threat thereof) against public policies or opponents |  | Passages were coded if they indicated that legal action had occurred or if threats of litigation were made |
| Opposition fragmentation and destabilization |  |  |  |
|  | Create multiple voices against public health measures |  | Passages were coded if they indicated that this had occurred |
|  | Criticize governmental and community organizations and advocates |  | Passages were coded if they criticized or portrayed government associations (e.g. Health Canada), public health organizations, public health advocates or other community groups in a negative light. Passages which criticized a *policy* or *proposal* were NOT coded; only direct criticism of *organizations* or *persons* was coded |
|  | Infiltrate, monitor and distract public health advocates, groups and organizations |  | Passages were coded if they indicated that this had occurred |
| Policy substitution |  |  |  |
|  | Develop and promote alternatives to proposed policies including revised policies, voluntary codes, self-regulation and non-regulatory initiatives |  | Passages were coded if they highlighted industry’s self-regulatory policies, voluntary codes and revisions to proposed government policies. When it was not clear if a certain initiative was voluntary or mandatory, the initiative was researched to determine if it was voluntary or mandated. Passages referring to industry compliance with mandatory policies were NOT coded |

**References**

1. Mialon M, Swinburn B, Sacks G. A proposed approach to systematically identify and monitor the corporate political activity of the food industry with respect to public health using publicly available information. Obesity Reviews. 2015;16(7):519-530.

**Table S1.** Number and percentage of each type of stakeholder in contact with Health Canada regarding the *Healthy Eating Strategy*, October 2016 to June 2018

| **Type of Stakeholder** | **n (%)** |
| --- | --- |
| Industry | 67 (52.8) |
| Advertising | 2 (1.6) |
| Food | 57 (44.9) |
| Sports | 2 (1.6) |
| Unspecified | 6 (4.7) |
| Non-Industry | 60 (47.2) |
| **Total** | 127 (100) |
